# Perovskite neural trees

**DOI:** 10.1038/s41467-020-16105-y

**Published:** 2020-05-07

**Authors:** Hai-Tian Zhang, Tae Joon Park, Ivan A. Zaluzhnyy, Qi Wang, Shakti Nagnath Wadekar, Sukriti Manna, Robert Andrawis, Peter O. Sprau, Yifei Sun, Zhen Zhang, Chengzi Huang, Hua Zhou, Zhan Zhang, Badri Narayanan, Gopalakrishnan Srinivasan, Nelson Hua, Evgeny Nazaretski, Xiaojing Huang, Hanfei Yan, Mingyuan Ge, Yong S. Chu, Mathew J. Cherukara, Martin V. Holt, Muthu Krishnamurthy, Oleg G. Shpyrko, Subramanian K.R.S. Sankaranarayanan, Alex Frano, Kaushik Roy, Shriram Ramanathan

**Affiliations:** 1 School of Materials Engineering, Purdue University, West Lafayette, IN 47907 USA; 2 Lillian Gilbreth Fellowship Program, College of Engineering, Purdue University, West Lafayette, IN 47907 USA; 3 Department of Physics, University of California, San Diego, La Jolla, CA 92093 USA; 4 School of Electrical and Computer Engineering, Purdue University, West Lafayette, IN 47907 USA; 5 Center for Nanoscale Materials, Argonne National Laboratory, Argonne, IL 60439 USA; 6 Department of Mechanical and Industrial Engineering, University of Illinois, Chicago, IL 60607 USA; 7 X-ray Science Division, Advanced Photon Source, Argonne National Laboratory, Lemont, IL 60439 USA; 8 Department of Mechanical Engineering, University of Louisville, Louisville, KY 40292 USA; 9 National Synchrotron Light Source II, Brookhaven National Laboratory, Upton, NY 11973 USA; 10 Department of Mathematics, University of Iowa, Iowa City, IA 52242 USA

**Keywords:** Materials for devices, Electronics, photonics and device physics

## Abstract

Trees are used by animals, humans and machines to classify information and make decisions. Natural tree structures displayed by synapses of the brain involves potentiation and depression capable of branching and is essential for survival and learning. Demonstration of such features in synthetic matter is challenging due to the need to host a complex energy landscape capable of learning, memory and electrical interrogation. We report experimental realization of tree-like conductance states at room temperature in strongly correlated perovskite nickelates by modulating proton distribution under high speed electric pulses. This demonstration represents physical realization of ultrametric trees, a concept from number theory applied to the study of spin glasses in physics that inspired early neural network theory dating almost forty years ago. We apply the tree-like memory features in spiking neural networks to demonstrate high fidelity object recognition, and in future can open new directions for neuromorphic computing and artificial intelligence.

## Abstract (web summary)

Designing energy efficient and scalable artificial networks for neuromorphic computing remains a challenge. Here, the authors demonstrate tree-like conductance states at room temperature in strongly correlated perovskite nickelates by modulating proton distribution under high speed electric pulses.

## Introduction

Artificial intelligence (AI) is poised to impact our society in a myriad of areas ranging from healthcare, synthesis of chemicals and fuels, to control systems of self-driving automobiles. Computers with powerful processors and memory together help usher this impending revolution. For AI to be sustainable, it is imperative to control the memory and thereby the intelligence and learning ability of autonomous machines. Creating new types of memory, including synaptic properties, is therefore a central goal in physical sciences and engineering research intersecting materials sciences, electronics, neuromorphic computers, and AI hardware^1–11^. In animal brains, evolution has enabled synapses (that are responsible for memory) to reach self-limiting weights as well as branching to avoid runaway effects and prevent catastrophic breakdown of neural circuits while still retaining the ability to learn throughout their lifespan. This remarkable synaptic weight update mechanism can be simply summarized as a tree structure. During a sequence of constant stimulus, the synaptic strength is modulated so that the weight of the synapse approaches self-limiting characteristics and maintains stability of the neural circuits^12,13^. At the same time, synapses preserve their capability to respond to a new stimulus^14–16^. Memory can be considered to be organized in a hierarchical way that can be represented in a tree structure^17,18^. Early neural network theories therefore incorporated tree-like states in their models to understand and emulate memory^18–23^. Low temperature magnetic states found in spin glasses such as CuMn arising from heating–cooling cycles in the 1–20 K temperature range have provided an experimental context^24,25^. Exploring the vast potential of tree-like states in several areas of neuromorphic computing continues to be an intensively studied topic in neural network theory^26,27^.

Here, we show that tree-like structures can be experimentally realized at room temperature in strongly correlated rare-earth perovskite nickelates (ReNiO_3_, where Re is a rare-earth ion), a class of quantum materials whose electrical properties are largely dominated by the strong interactions among electrons in them^28–30^. For example, in NdNiO_3_ and SmNiO_3_, prior studies have shown that hydrogen doping results in several orders of magnitude change in electrical resistance via modifying electron occupancy of the Ni orbitals^31,32^. When hydrogen molecules are split by a catalytic electrode into hydrogen atoms and doped into the rare-earth perovskite nickelate lattice, the electron from the hydrogen atom is injected to the Ni e_g_ orbital, while the proton resides in interstitial sites. The electron injection changes the filling state of the Ni d band and opens up a large transport gap due to a Mott transition. Synaptic potentiation and depression studies have been reported with nickelates in the millisecond timescales with this strategy^32^. Proton migration-driven organic systems have also been reported as artificial synapses suggesting their broad relevance in emerging memory devices^33^.

## Results

### Conceptual description of the neural tree

**Fig. 1:**
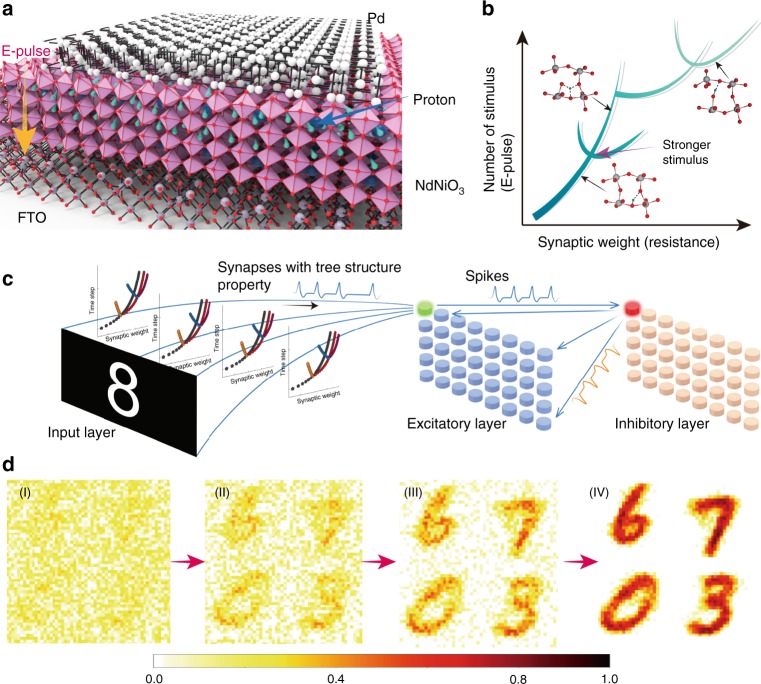
Perovskite neural trees and their use in neuromorphic learning. **a** Schematic figure of the perovskite nickelate NdNiO_3_ device with Pd as top electrode and fluorine-doped tin oxide (FTO) as bottom electrode. The top electrode serves also to catalytically dope hydrogen into the near-surface region of the perovskite. Applying electric field pulses can move the protons in the lattice which also changes the local Ni valence state and electron-electron correlation, thus modulating the device resistance in a systematic manner. **b** Schematic of the tree structure showing synaptic strength (resistance) as function of number of stimulus (electric pulses). The electrical resistance of the perovskite devices can be modulated with consecutive electric pulses. The snapshots schematically show the movement of protons in the lattice, which leads to different resistivity values. **c** Architecture of spiking neural network for handwritten digit recognition. Each input image pixel is assigned to one input neuron. Input layer generates Poisson’s distributed spike train depending on the pixel intensity values, which potentiates the membrane potential (*V*_mem_) of excitatory layer neuron. These spikes are propagated from input to excitatory layer through synapses which learns using spike time dependent plasticity (STDP) learning rule. Once membrane potential reaches a threshold (*V*_thresh_), the neuron generates a spike and synapse weights are updated. The tree structure graph represents how synaptic weight changes with input strength. Different curves correspond to different constant inputs. **d** Evolution of digit learning using the tree-like synapses. Step (I) shows the synapse weights at initial stages of learning, (II) and (III) show weights after learning from 10,000 and 30,000 training images, and (IV) is the final learned weights after training on 60,000 images.

We demonstrate that electric pulses, as fast as tens of nano seconds, are effective in perturbing the proton distribution in the nickelate lattice and can tune its resistivity in a systematic fashion enabling ultrametric tree-like conductance states, as schematically shown in Fig. 1a. By applying electric pulses in consecutive and reversible manner, a tree structure of memory states can be generated as a function of number of pulses, as schematically shown in Fig. 1b. The weight control factors are computed from the tree structure after we apply an input, as shown in Fig. 1c. The tree-structured algorithmic weight changes as a function of time steps are then used for simulation in a spiking neural network (SNN) as synapses between the input layer and the excitatory layer to demonstrate proof-of-concept application in learning using MNIST digits, a widely used database by the neuromorphic engineering community (see Methods section and Supplement Note 1 for details). A typical learning evolution process with such SNN for handwritten digit recognition is shown in Fig. 1d.

### Experimental ultrametric memory trees

**Fig. 2:**
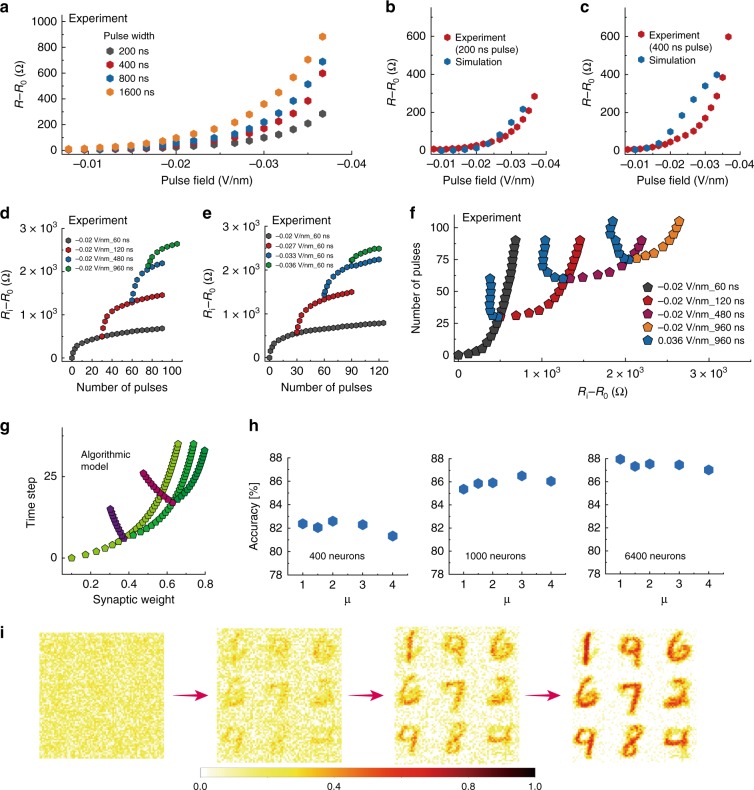
Experimental data of electrical behavior and simulation in neural networks. **a** Change in electrical resistance of the nickelate device after voltage pulses were applied with different pulse field and pulse width. The change in resistance is proportional to both the pulse field and pulse width. **b**, **c** Modeling and experimental results of the change in resistance after application of various voltage pulses, **b** 200 ns pulse, and **c** 400 ns pulse widths. **d**, **e** The electrical response of the nickelate device to consecutive electrical pulses. The different colors in (**d**) represent different pulse widths, while the different colors in (**e**) represent different pulse field. The structure was generated following the method shown in Supplementary Fig. 6. **f** By applying consecutive positive or negative pulses, tree structure comprising different resistance states can be generated, with characteristics similar to synaptic behavior in the central nervous system^12,14–16^. The same colored data points correspond to a fixed input that is applied to the device. **g** Algorithmic weight change as a function of learning time steps. The synaptic weight update mechanism has a tree structure that is inherently possessed by the nickelate devices (for *μ* = 3). **h** Object recognition testing accuracy of a spiking neural network on Modified National Institute of Standards and Technology database (MNIST) dataset when trained with different *μ* values on 400, 1000, and 6400 excitatory neurons. Testing accuracy change of about ±0.5% is observed, which does not significantly hamper the network’s digit recognition performance, hence the network is sufficiently immune to variations in *μ*. **i** Weight evolution of nine neurons when the spiking neural network is trained using spike time dependent plasticity (STDP) learning rule. The learning evolves such that the weights associated with each neuron approximates one of the variations of the digit learned by that neuron since each neuron in excitatory layer is connected to all neurons of the input layer.

The experimentally measured electrical properties of the hydrogen-doped nickelate devices and their tree-like synaptic behaviors are summarized in Fig. 2. Positive voltage sweeps were first applied, and gradual decrease of device resistance was observed, while the resistance increased when negative sweeps were applied (see Supplementary Fig. 3). The response to electrical pulses of different pulse field and pulse width are shown in Fig. 2a. The increase in resistance was found to be proportional to both magnitude of the pulse field and pulse width consistent with the expectation for proton migration under a driving force. The memory state is non-volatile as shown in Supplementary Fig. 4. As expected, no response was observed if identical electrical pulses (or even stronger pulses) were applied to control samples of undoped pristine nickelate devices, see Supplementary Fig. 5. By solving a set of electro-thermal equations self-consistently (see Methods section for details), we simulate the proton drift under electrical pulses. Since the resistance of the nickelate device is exponentially related to the local proton concentration^34^, a positive electric pulse will dilute the proton concentration near the Pd electrode and thus decrease the device resistance and vice versa. The simulated total device resistance under different pulse fields and pulse widths is plotted in Fig. 2b, c, which is consistent with the experimentally measured data.

We then demonstrate the weight modulation of the nickelate device by applying consecutive electric pulses (Fig. 2d). The resistance of the nickelate device can be modulated by an electric pulse of magnitude as low as 0.02 V/nm, 60-ns duration (black curve), and the device resistance increases under consecutive constant inputs. During this process, a weight control mechanism was observed that inherently decreases the slope of synaptic strength change with increasing number of pulses. Applying a larger stimulus, however, can reactivate the updating process and generate a distinct resistance branch, e.g., see the red curve in Fig. 2d and Supplementary Fig. 6 for details in generating a new branch. This trend is akin to synaptic strength updates in animal brains, which maintains the stability of the neural circuits to avoid hyper-activity^12,13^. Controlled synaptic weight updating mechanism was observed for multiple pulse widths, as shown in Supplementary Fig. 7 and is an inherent feature of our proton migration-driven devices. Natural synapses also preserve the ability to respond to larger stimulus^14–16^. Similarly here, new branches can be generated by further increasing the pulse width (blue and green curve in Fig. 2d) or the pulse field (see Fig. 2e). By applying positive or negative electrical pulses, a tree structure with different memory states can be generated, as shown in Fig. 2f and Supplementary Fig. 8 for more sophisticated neural tree structures. A unique combination of atomic-level control over proton migration in nickelates under high speed e-fields coupled with their ultra-sensitivity to Ni-site orbital occupancy is responsible for generation of the tree-like memory. Our experimentally measured trees can be classified according to number theory as ultrametric (see Supplement Note 2 for a mathematical description) and represent physical realization of a mathematical concept considered critical for solving a myriad of problems in neural computing^26^.

### Application of the tree-like conductance in spiking neural networks

As proof of principle, we utilize the tree-like synaptic behavior of nickelates into spiking neural networks using spike time dependent plasticity (STDP) as an unsupervised learning rule. We track the spiking activity of neurons by maintaining spike trace of pre-synaptic neuron (*x*_pre_). The trace value is updated by 1 whenever the neuron spikes and decays exponentially along the time steps. As shown in Eq. (1), depending on the threshold trace value (*x*_tar_), we determine whether we potentiate or depress the synapse strength (weight). If (*x*_pre_ – *x*_tar_) is positive, Δ*ω* is positive hence potentiation occurs and when (*x*_pre_ – *x*_tar_) is negative, Δ*ω* is negative hence depression of synaptic strength (weight).

1 $$\Delta {\upomega} = {\upeta}({x_{{\mathrm{pre}}} - x_{{\mathrm{tar}}}})( {{\upomega }}_{{\mathrm{max}} - {\upomega}})^{\upmu}$$

*ω*_max_ is the maximum weight the synapse can attain, *ω* is the current weight of the synapse and *μ* controls the rate of change. Figure 2g represents algorithmic weight change curve which mimics the experimental tree structure of the nickelate device. Each curve shows weight change as an input that is applied over time (different colors represent different input magnitude). 400, 1000, and 6400 neurons were trained and tested (see Fig. 2h). The network achieves accuracy of 82.9%, 86.5%, and 87.4% for the network with 400, 1000, and 6400 neurons for *μ* = 3 that most closely fits the controlled weight updating mechanism of our nickelate devices, see Supplementary Fig. 9. The digit recognition accuracy is robust to changes in *μ* and only changes within an average margin of ±0.5%. Hence the performance of the network is robust to small variations in *μ* that could arise from any fabrication process variations. Figure 2i shows synapse strength (weight) evolution during learning for *μ* = 3 of a device abstracted to the algorithm. The graph shows synaptic weights of nine neurons. Each excitatory neuron is fully connected to input layer neurons; therefore, the learned synaptic weights imitates the digit learned by that neuron for instance, 1st neuron has learned a particular variation of digit 1, 2nd neuron has learned digit 9, 3rd and 4th has learned 6.

### Microscopic origins of tree-like memory states

**Fig. 3:**
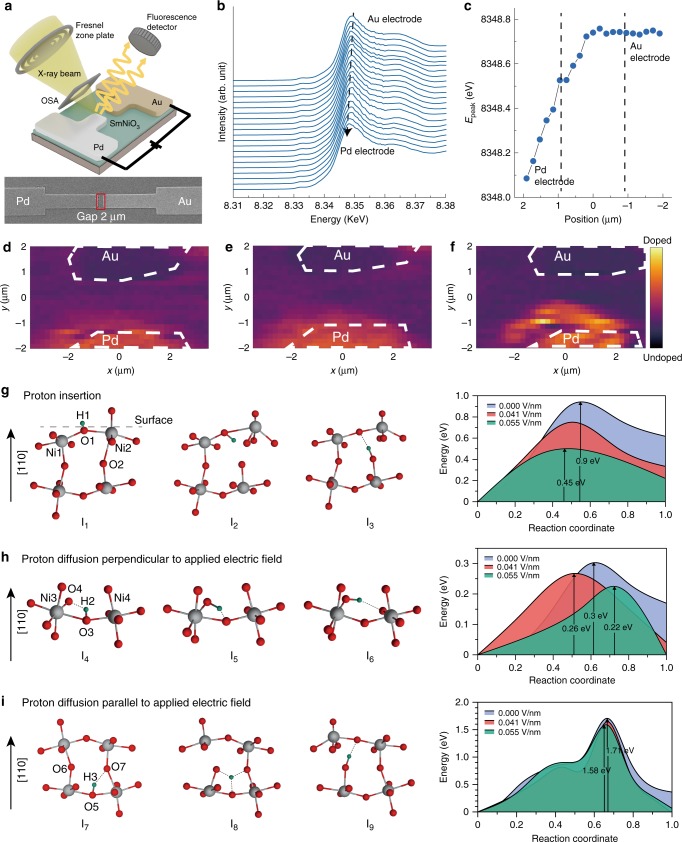
Microscopic mechanism leading to the tree-like synaptic memory. **a** Scheme of the nanoprobe X-ray absorption imaging experiment. The incoming X-ray beam is focused by the Fresnel zone plate, and diffraction orders are filtered out by the order-sorting aperture (OSA). The fluorescence signal at the K-edge of Ni from the 30-nm spot illuminated by the X-rays is recorded by the detector positioned perpendicular to the beam. A scanning electron microscope (SEM) image of the nickelate device is shown at the bottom. The red rectangle shows the scanned area of the X-ray absorption imaging. **b** Changes in the XAS spectrum at Ni K-edge as the probe is rastered across the device channel. The spectra were measured at different positions between the electrodes and averaged along the electrode edge, starting from the Pd electrode (the lowest spectrum) to the Au electrode (the upper spectrum). The distance between two successive spectra is Δ*y*  =  200  nm. The dashed line is shown for better visualization of the peak shift. From the Au electrode to the Pd electrode, the Ni K-edge peak shifts to lower energy, indicating the change in Ni valence and proton doping near the Pd electrode. **c** The fitted energy value of the Ni K-edge peak plotted as function of sample position. The fitting error is ~0.15  eV which was calculated by using different fitting procedures and evaluating the distribution of the obtained values of E_peak_. **d**, **e**, **f** Changes in Ni K-edge fluorescence intensity of the hydrogenated nickelate device after electrical stimuli. The color corresponds to the intensity of the normalized fluorescence signal at the fixed energy of *E*  =  8345  eV (where the slope of the XAS curve is highest and thus most sensitive to the energy shift) and therefore characterizes the hydrogen doping of the channel. Dashed lines denote the positions of the Au and Pd electrodes. The map of the initial sample is shown in panel (**d**), the map after applying several 2 mV pulses of 5 -s duration time is shown in (**e**), and after several 1  V pulses of 100- ns duration in (**f**). **g** Atomic-scale pathway, and the associated energy barriers for various applied e-fields for surface proton doping into the nickelate lattice. The potential energy along the most preferred migration pathway (as obtained from nudged-elastic band (NEB) DFT calculations) is shown on the left, while selected configurations along this pathway labeled I_1_–I_3_ are depicted on the right. A barrier of 0.9 eV was obtained for this depicted proton intercalation pathway with no field. Electric fields reduce the barrier—a 50% reduction in barrier is seen for an applied e-field of 0.055  V/nm. Two different pathways for proton diffusion in the bulk are shown. Panel (**h**) represents a lower (preferred) pathway, whereas (**i**) represents a high energy barrier for bulk diffusion in the presence of the e-field. For all the depicted configurations, only two NiO_6_ octahedra are shown for clarity; the Ni, O, and H ions are represented by gray, red, and green spheres, respectively.

To understand the microscopic origins of tree-like memory in the hydrogen-doped nickelates, we performed in operando X-ray studies of a representative in-plane nickelate device. For this experiment, a perovskite nickelate SmNiO_3_ in-plane device is catalytically doped with protons using a Pd electrode on top of the film through annealing in forming gas. We then apply electrical stimuli to the nickelate device and track the changes in the electronic structure by measuring X-ray absorption spectra (XAS) maps, and a schematic of the setup is shown in Fig. 3a. The incident X-ray beam focused by a Fresnel zone plate to ~30 nm is used to perform the raster scan of the device between the electrodes with steps of 200 nm. The XAS at K-edge of Ni (*E* ~ 8347 eV) collected at different positions between the Pd and Au electrodes are shown in Fig. 3b. The Ni K-edge position proximal to the Au electrode is similar to previous reports on SmNiO_3_^35,36^, and the shift of the Ni K-edge energy by ~0.6 eV is clearly visible on the XAS spectra measured next to Pd as compared with the regions away from the electrode (see Fig. 3c). This shift can be attributed to the formal decrease of the Ni valence by a small amount *δ* due to the additional electrons introduced by hydrogen doping^37,38^, which agrees with the calibration experiment performed on a 10 mm × 10 mm nickelate film (Supplementary Fig. 10). The decrease of the Ni-ion valence is noticeable directly underneath the Pd electrode and it spans up to a distance of several hundred nanometers away from the electrode determined by hydrogen diffusion during doping.

To reveal the spatial distribution of the hydrogen-doped areas, we fixed the energy of incoming X-ray photons at *E* = 8345 eV, which corresponds to the maximum of the Ni absorption spectrum derivative and where the shift of the absorption edge is most visible. In this case, the areas of the nickelate device doped by hydrogen are distinguishable by a more intense fluorescence signal due to the shift of the Ni K-edge toward lower energies. The normalized fluorescence map of the as-hydrogenated device is shown in Fig. 3d. The yellow areas next to the Pd electrode indicate the regions with a reduced valence of Ni, while the purple areas farther away from the Pd electrode correspond to the unchanged electronic structure of pristine SmNiO_3_, see Supplementary Fig. 11 for the calibration of electrode position. To check whether the electric pulses lead to the changes in the nickelate electronic structure, we tested two different types of electrical stimuli and collected similar fluorescence maps. First, we applied several long-duration pulses (5 s) with 2 mV magnitude. The fluorescence map after this type of stimulus (Fig. 3e) reveals only little changes compared with the initial state of the device. Subsequently, shorter 100-ns pulses of 1 V magnitude were applied to the device, which caused significant changes in the fluorescence map (Fig. 3f). The non-uniform distribution of the fluorescence intensity with typical length scale of 1 μm appears next to the Pd electrode—the region initially doped with hydrogen. Although it is extremely challenging to detect protons directly in nanoscale regions in a solid matrix, the electronic structure spatial maps presented here demonstrates that voltage pulses can cause migration of protons in the nickelate device from tracking the reconfiguration of Ni charge states.

The effect of electric field on activation barrier associated with proton migration was derived using nudged-elastic band (NEB) calculations based on density functional theory (DFT) with Hubbard corrections. The H diffusion pathways (left panel) and associated barriers (right panel) are shown in Fig. 3g–i. Figure 3g shows the e-field effect on surface insertion of proton into the SmNiO_3_ (110) lattice. Proton labeled as H is initially attached to the surface oxygen O1 (image I_1_) that is shared by two NiO_6_ octahedra centered at nickel atoms Ni1 and Ni2. The distance between O1 and the two nickel ions (Ni1 and Ni2) are 1.99 and 2.15 Å, respectively. The proton H rotates enters into the subsurface layer of SmNiO_3_ via rotation of O1–H bond (refer to image I_2_), causing the O2–H separation to reduce from 3.72 to 2.12 Å. Further rotation (of O1–H) and consequent displacement of H toward O2 eventually results in the formation of O2–H bond; thereby causing H to hop from surface O1 and subsurface O2. The energy barrier associated with this H-hopping pathway is ~0.9 eV with no field. With electric field, this barrier reduces by 22% for *E* = 0.04 V/nm and by 50% for *E* = 0.055 V/nm.

We investigated the e-field effect on two different bulk diffusion pathways (see Fig. 3h, i). At first, proton hopping occurs perpendicular to [110] with the field applied along [110] direction. Here, the proton (H2) is initially bonded to the oxygen (O3) (image I_4_). The oxygen O3 is also shared by two NiO_6_ octahedra centered at Ni3 and Ni4. O3 is located deep inside SmNiO_3_ lattice (i.e., not at surface or subsurface of SmNiO_3_ lattice). In this diffusion pathway, the proton H2 moves toward oxygen O4 and creates a bond O4–H2 (image I_5_); note in I_5_, O4–H bond is ~18% longer than typical O–H bonds. Further motion of H along the pathway brings it closer to O4 with concomitant reduction of Ni3–O3–Ni4 bond angle 156° to 146°. The barrier of this pathway is 0.3 eV in absence of electric field. For an applied electric field (*E* = 0.055 V/nm) normal to the hopping direction, we observe a lowering in the barrier (by ~80 meV). Next, we choose a bulk diffusion pathway where the hopping is aligned with the direction of the applied e-field. Here, the hydrogen H3 is initially connected to oxygen O5 as shown in image I_7_. H3 moves into a transition state such that it maintains approximately equal distance of 1.5 Å from three different oxygens O5, O6, and O7 (refer to image I_8_). Subsequently, it moves further upward resulting in a slight rotation of the O6–H3 bond. This pathway is associated with a barrier of 1.7 eV when no e-field is applied, which is much higher compared with that for the aforementioned pathways. Similar to other pathways, e-field reduces the barrier for this path (by ~130 meV). Our NEB calculations suggest that the application of e-field lowers the activation barrier for H diffusion in SmNiO_3_ lattice in all scenarios. However, the extent of barrier-reduction and therefore the proton migration characteristics are influenced by the magnitude of the applied field similar to our experiments. In the main article (Fig. 3g, h, i), we have described effect of electric field on proton diffusion by calculating the activation barrier associated with the proton migration. The detailed pathways of proton migration for Fig. 3g, h, and i are described in Supplementary Fig. 12.

## Discussion

In Supplementary Fig. 13, we demonstrate tree-like synaptic states measured from nickelate devices fabricated on silicon substrate to illustrate proof-of-concept compatibility with existing semiconductor platforms. In-plane nickelate devices with 100 nm gap size were also fabricated and studied to examine scalability, as shown in Supplementary Fig. 14. The modulation of the nickelate resistivity is found to be reproducible which enables tracing resistance paths to create multiple branches upon application of voltage pulses. We can also estimate the energy consumption during weight update by ${\mathrm{Energy}} = {\it{V}}^2{\it{t}}/R$, where *V* is the pulse voltage, *t* is the pulse width and *R* is the device resistance. The energy cost is ~2.7 × 10^−11^ J/pulse which is comparable to other types of oxide resistive switching devices reported in literature^39–41^.

In conclusion, we have demonstrated ultrametric tree-like memory states in hydrogen-doped perovskite nickelates controlled by pulsed electric fields at room temperature. Controlled ion migration that can synergistically mediate highly tunable electronic structures in correlated perovskite crystals enable realization of a set of properties previously used in description of spin glasses. The results should motivate new avenues to explore use of complex synaptic behavior found in correlated semiconductors for neuromorphic learning.

## Methods

### NdNiO_3_ and SmNiO_3_ thin film synthesis

NdNiO_3_ and SmNiO_3_ films (150 and 100 nm calibrated by profilometer, respectively) were deposited with a high vacuum sputtering system from AJA International, Inc. The NdNiO_3_ films were grown by sputtering a NdNiO_3_ ceramic target with radio frequency (RF) power at 150 W at room temperature. During deposition, the background pressure was set at 10 mTorr of Ar/O_2_ mixture at 4:1 ratio. The film was then annealed at 500 °C for 24 h in ambient atmosphere. For the NdNiO_3_ film grown on silicon, 100 nm indium tin oxide (ITO) was first deposited using e-beam evaporation on the silicon substrate as bottom electrode before the NdNiO_3_ film was deposited. The SmNiO_3_ films were grown in the system with co-depositing Sm and Ni targets at room temperature, with a RF power at 170 W for Sm and a direct current (DC) power at 70 W for Ni. The growth condition was calibrated by energy-dispersive X-ray spectroscopy to ensure the cation stoichiometry. The growth background pressure was 5 mTorr of Ar/O_2_ mixture at 4:1 ratio, and then high oxygen pressure annealing (100 atmosphere) was performed at 500 °C for 24 h in a tube furnace.

### Vertical and in-plane device fabrication

For the vertical devices, the annealed films were first rinsed with Isopropyl alcohol (IPA) and then covered with shadow mask for the Pd electrode deposition. The Pd electrodes were 150 μm in radius and 50-nm thick, which were deposited at room temperature using sputtering under 100 W DC power and 5 mTorr Ar background pressure. Before fabrication of the in-plane device, the sample was ultrasonic cleaned by toluene, acetone and IPA for 5 min each. To avoid shorting between two terminals, the SmNiO_3_ film was etched by ion mill, with photo resist SPR-220-3.5 as a mask layer. The photo resist was removed by acetone and PG-remover at 80 °C. The first lithography (150 nm Au) was carried out, followed by the e-beam evaporation and lift-off in PG-Remover at 80 °C. To maintain the high resolution of patterns as well as the accuracy of the small gap distance in between Au and Pd electrodes, e-beam lithography was utilized. Based on the alignment marks in the pattern, the first aligned e-beam lithography was able to expose the Au electrodes. After 50 nm Au deposition and lift-off by acetone at room temperature, the second aligned e-beam lithography exposed for small Pd electrodes and a 50 nm Pd layer was deposited by e-beam evaporation. After lift-off as well as cleaning, the left-connection electrodes (150 nm Pd) was deposited, in the same way as the right-connection electrode deposition. Finally, wire-bonding pads (5 nm Ti/150 nm Au) were deposited by photo lithography and e-beam evaporation. The photo resists used in electrode deposition process were LOR-3A and S1813. The e-beam resists used in e-beam lithography were 495 PMMA A4 and 950 PMMA A4. The fabrication process of the vertical device and in-plane device are shown in Extended Fig. 1 and Extended Fig. 2, respectively. For hydrogen incorporation, Pd electrodes served as catalysts to split hydrogen molecules. The vertical devices were annealed in 50 SCCM H_2_/N_2_ (5%/95%) gas at 300 °C for 5 min, and the in-plane devices were annealed in 50 SCCM H_2_/N_2_ (5%/95%) gas at 120 °C for 5 min.

### Electrical measurements

Electrical measurements of the devices were performed at room temperature and a Micromanipulator probe station was used to probe the devices. The probe station was located on a 63-2405X vibration isolation table to minimize noise due to vibration. A Keithley 4200A-SCS Ultra-fast pulse measure units (4225-PMU) and remote preamplifier/switch modules (4225-RPM) were used to provide nano-second pulses and ultra-low noised triax cables were used to minimize noise during the measurement. The device resistance was obtained by fitting the voltage–current curve in the linear region (from −0.01 to 0.01 V). The measurements were controlled through the Clarius program designed by Tektronix. All the electric fields applied are referenced to the Pd electrode.

### Simulation methodology for SNN

An open-source spiking neural network simulator (BRIAN) was used^42^. We used the framework provided by Diehl et al. for MNIST digit recognition application in python3 since the original framework was in python2^43,44^. Neuron model was updated from a conductance based model (complete description can be found in the Supplement file) which has direct control over the potential of neuron when a spike arrives at a post-synaptic neuron from one or more pre-synaptic neurons. The synapse weight update equation was modified to a STDP learning rule which only updates weight when a post-synaptic neuron fires and spiking activity trace of a pre-synaptic neuron is compared against a pre-synaptic threshold to determine the amount of potentiation or depression of synaptic weight.

### Ab initio molecular dynamics (AIMD) simulations

The first principles calculations were performed within DFT + *U* approximation using VASP^45^ at the level of generalized gradient approximation using the Perdew-Burke-Ernzenhof exchange-correlation functional^46^. We have used projector-augmented pseudopotentials^47^, Sm_3 (valence 5 s 5p2 6s 4f), Ni_pv (valence 3p6 4s 3d), O (valence 2s2 2p4), and H (valence 1s1), and the rotationally invariant form of DFT + *U* from ref. ^48^ with *U* = 4.6 eV and *J* = 0.6 eV, where *U* is the on-site Coulomb parameter and *J* is the on-site exchange parameter. The Brillouin zone was sampled at the Γ-point only and the planewave cutoff energy was set to 540 eV in all calculations. The energy tolerance stopping criteria were 10^−5^ and 10^−4^ for the electronic and ionic convergence, respectively. For the crystal structure prediction of pure SmNiO_3_, we started with the structure collected from Materials Project database^49^. We added a small monoclinic distortion (*β* ≈ 90.75) and allowed the cell and ionic positions to relax until we met the electronic and ionic convergence criterion. The H SmNiO_3_ structures were adopted by AIMD simulations from literature^50,51^. To compute the barrier associated with H migration on SmNiO_3_ [110] slab, we used climbing image nudged-elastic band method. A 35-Å-thick vacuum region is introduced to separate the periodic slabs along the z-direction. Simulations were performed with and without e-field. The external electric field, with magnitudes up to 0.055 V/nm, were applied perpendicular to the plane of the HSmNiO_3_ layer (along the z-direction of the cell). Spin-polarization was introduced, as well as a mixing scheme (AMIX = 0.1, BMIX = 0.001) was used in order to facilitate the convergence of electronic states.

### Simulation of proton migration under electric fields

The resistance of the nickelate devices is modulated by the spatial concentration of hydrogen ions. Hydrogen donates an electron to the nickel site changing its valence to 2+ and forming a proton in the interstitial site. The 2+ Ni-site is heavily insulating and thereby localizes charge carriers. The distribution of the protons in the channel therefore controls the overall resistance. The resistivity of the nickelate device can be defined as follows^34^

2 $${\it{\uprho }} = \frac{{{\it{\uprho }}_0}}{{\left( {1 + {\mathrm{e}}^{\frac{{{\it{n}}_0 - {\it{n}}}}{{{\it{n}}_{\mathrm{d}}}}}} \right)}}{\mathrm{,}}$$

where *n* is the concentration of Ni^2+^, *ρ*_0_, *n*_0_, and *n*_d_ are fitting parameters. The exponential relation between concentration of Ni^2+^ and resistivity reflects that the channel resistance is limited by the high proton-concentration region near the upper electrode. In the case of positive gate voltage, the hydrogen atoms migrate from the high-concentration region near the upper electrode toward the dilute regions near the bottom electrode. The peak proton-concentration decreases and the dilute region concentration increases. Consequently, the resistance of the high-concentration region decreases and dilute region resistance increases according to Eq. (2).

The proton motion induced by the external potential is formulated by the drift-diffusion equation as follows^52^:

3 $$\frac{{\partial n}}{{\partial t}} = - \nabla .vn,$$

where *D* is the diffusion coefficient, and *v* is the drift velocity that is defined as^53^

4 $${\it{v}} = {\it{aw}}_{\it{{\mathrm{d}}}}{\it{fe}}^{ - \frac{{{\it{E}}_{\mathrm{a}}}}{{{\it{k}}_{\mathrm{B}}{\it{T}}}}}{\mathrm{sinh}}\left( {\frac{{{\it{qaE}}}}{{{\it{k}}_{\mathrm{B}}{\it{T}}}}} \right){\mathrm{,}}$$

where *E* is the electric field, *q* is the electron charge, *a* is the hopping distance, *k*_B_ is the Boltzmann’s constant, *T* is the temperature, *w*_d_ is the window function, *f* is the attempt frequency, and *E*_a_ is the diffusion barrier. The temperature of the channel has a strong influence on the drift velocity as descried by the exponential factor in Eq. (4). The channel temperature could be calculated by solving the boundary value problem defined by the Fourier equation.

5 $$- \nabla .{\it{k}}_{{\mathrm{th}}}\nabla {\it{T}} = \frac{{\left| {\nabla {\mathrm{\Psi }}} \right|^2}}{{\it{\uprho }}},$$

where *k*_th_ is the thermal conductivity, and $\Psi$ is the electric potential. $\frac{{\left| {\nabla {\mathrm{\Psi }}} \right|^{\mathrm{2}}}}{{\it{\uprho }}}$ is the Joule heating term. The ambient temperature is assumed as a boundary condition at the two electrodes. The window function *w*_d_ is defined as follows:

6 $${\it{w}}_{\mathrm{d}} = 1 - \left( {\left( {\frac{{\it{x}}}{{\it{L}}} - 0.5} \right)^2 + 0.75} \right)^{2{\it{p}}}$$

where *L* is the length of the channel, and the exponent *p* defines the steepness of the window function at the electrodes. The window function emulates the non-linear drift effect at the boundaries of the channel. Finally, the electric potential and the electric field are calculated by solving the current continuity equation

7 $$\nabla .\frac{1}{{\it{\uprho }}}\nabla \Psi = 0.$$

The parameters used in the simulation are *f* = 0.7815 × 10^12^ s^−1^, *a* = 0.43478 nm, *E*_a_ = 0.26 eV, *k*_th_= 3 Wm^−1^ K^−1^,^54^ *ρ*_0_ = 8.9578 × 10^4^ $\Omega$. cm, *n*_0_ = 0.73, *n*_d_ = 0.12, and *p* = 3.

### In situ nanoprobe X-ray absorption spectroscopy measurements (Brookhaven National Laboratory, Argonne National Laboratory)

In operando X-ray studies of the in-plane nickelate device at the 3-ID Hard X-ray Nanoprobe (HXN) Beamline of the National Synchrotron Light Source II and the 26-ID Hard X-ray Nanoprobe Beamline of the Advanced Photon Source^55–57^. The device for the X-ray experiment consisted of 100-nm-thick SmNiO_3_ film on (111)-oriented LaAlO_3_ substrate. On top of the film we deposited two rectangular 5-μm-wide electrodes of Au and Pd with the 2 μm gap between them to dope the film with hydrogen and apply electrical pulses. The XAS spectra were collected with 0.5 eV resolution near the Ni K-edge. At each energy, the device was scanned by the focused X-ray beam with 200 nm step in the area 4 × 7 μm^2^ (vert. × hor.) around the gap. The footprint of the X-ray beam on the sample was ~30 nm × 170 nm (vert. × hor.). The fluorescence signal from Au and Pd electrodes was used to align the device before each scan. The position of the Ni K-edge absorption peak *E*_peak_ was determined by fitting the experimental data at *E* = 8340–8350 eV with a sum of the Gaussian and linear functions. Each XAS spectra used to reconstruct the maps was initially normalized to its maximum value in order to exclude the possible effects of the film thickness on intensity of the fluorescence signal.

### X-ray absorption spectroscopy measurements (Argonne National Laboratory)

Additional XAS measurements were carried out at the station 33-ID-D at Advanced Photon Source. The sample was mounted at the center of a 6-circle diffractometer with a PilatusII-100K detector collecting the scattered X-ray intensity and a Vortex detector collecting the fluorescence signal simultaneously. X-ray energy was scanned through Ni K-edge between 8.31 and 8.56 keV. The incidence-angle of the measurement is around 5.2° with penetration depth of beam >100 nm. The measurement area is around 0.5 mm × 0.5 mm. All spectra were measured at room temperature.

## Supplementary information

Supplementary Information

## Data Availability

The data that support the findings of this study are available from the corresponding author upon reasonable request.

## References

[1] Burr GW (2017). Neuromorphic computing using non-volatile memory. Adv. Phys. X.

[2] Wang Y (2019). Mott-transition-based RRAM. Mater. Today.

[3] Guo Y, Wu H, Gao B, Qian H (2019). Unsupervised learning on resistive memory array based spiking neural networks. Front. Neurosci..

[4] Zhou Y, Ramanathan S (2015). Mott memory and neuromorphic devices. Proc. IEEE.

[5] Andrews JL, Santos DA, Meyyappan M, Williams RS, Banerjee S (2019). Building brain-inspired logic circuits from dynamically switchable transition-metal oxides. Trends Chem..

[6] Strukov DB, Kohlstedt H (2012). Resistive switching phenomena in thin films: materials, devices, and applications. MRS Bull..

[7] Keim NC, Paulsen JD, Zeravcic Z, Sastry S, Nagel SR (2019). Memory formation in matter. Rev. Mod. Phys..

[8] Fuller EJ (2019). Redox transistors for neuromorphic computing. IBM J. Res. Dev..

[9] Kuzum D, Yu S, Wong H-SP (2013). Synaptic electronics: materials, devices and applications. Nanotechnology.

[10] Serb A (2016). Unsupervised learning in probabilistic neural networks with multi-state metal-oxide memristive synapses. Nat. Commun..

[11] Saïghi S (2015). Plasticity in memristive devices for spiking neural networks. Front. Neurosci..

[12] Turrigiano GG, Nelson SB (2004). Homeostatic plasticity in the developing nervous system. Nat. Rev. Neurosci..

[13] Zhuang X (2001). Hyperactivity and impaired response habituation in hyperdopaminergic mice. Proc. Natl Acad. Sci. USA.

[14] Irie T, Ohmori H (2008). Presynaptic GABAB receptors modulate synaptic facilitation and depression at distinct synapses in fusiform cells of mouse dorsal cochlear nucleus. Biochem. Biophys. Res. Commun..

[15] Yamamoto, K., Noguchi, J., Yamada, C., Watabe, A. M. & Kato, F. Distinct target cell-dependent forms of short-term plasticity of the central visceral afferent synapses of the rat. *BMC Neurosci*. **11**, 134 (2010). 10.1186/1471-2202-11-134 PMC2978217 20961403

[16] Durand D, Carlen PL (1984). Impairment of long-term potentiation in rat hippocampus following chronic ethanol treatment. Brain Res..

[17] Feigel’man, M. V. & Ioffe, L. B. in *Models of Neural Networks I. Physics of Neural Networks* (eds E., D., J.L., van H. & K., S.) 181–200 (Springer, 1991).

[18] Sakaguchi H (1989). A hierarchical neural network model for category detection. Prog. Theor. Phys..

[19] Dotsenko, V. *An Introduction to the Theory of Spin Glasses and Neural Networks*. *World Scientific Lecture Notes in Physics* Vol. 54 (WORLD SCIENTIFIC, 1995).

[20] Willcox CR (1989). Exponential storage and retrieval in hierarchical neural networks. J. Phys. A. Math. Gen..

[21] Cortes C, Krogh A, Hertz JA (1987). Hierarchical associative networks. J. Phys. A Gen. Phys..

[22] Van Hemmen JL (1985). Spin-glass models of a neural network. Phys. Rev. A.

[23] Amit DJ, Gutfreund H, Sompolinsky H (1985). Storing infinite numbers of patterns in a spin-glass model of neural networks. Phys. Rev. Lett..

[24] Suzuki IS, Suzuki M (2006). Effect of random disorder and spin frustration on the reentrant spin-glass and ferromagnetic phases in the stage-2 Cu_0.93_Co_0.07_Cl_2_ graphite intercalation compound near the multicritical point. Phys. Rev. B.

[25] Nagata S, Keesom PH, Harrison HR (1979). Low-dc-field susceptibility of CuMn spin glass. Phys. Rev. B.

[26] Hartnett GS, Parker E, Geist E (2018). Replica symmetry breaking in bipartite spin glasses and neural networks. Phys. Rev. E.

[27] Hartnett, G. S. & Mohseni, M. Self-supervised learning of generative spin-glasses with normalizing flows. Preprint at http://arxiv.org/abs/2001.00585 (2020).

[28] Catalan G (2008). Progress in perovskite nickelate research. Phase Transit..

[29] Catalano S (2018). Rare-earth nickelates RNiO_3_: thin films and heterostructures. Rep. Prog. Phys..

[30] Keimer B, Maier J, Mannhart J (2012). Electronic materials through time. Nat. Mater..

[31] Oh C, Jo M, Son J (2019). All-solid-state synaptic transistors with high-temperature stability using proton pump gating of strongly correlated materials. ACS Appl. Mater. Interfaces.

[32] Ramadoss K (2018). Proton-doped strongly correlated perovskite nickelate memory devices. IEEE Electron Device Lett..

[33] Van De Burgt Y (2017). A non-volatile organic electrochemical device as a low-voltage artificial synapse for neuromorphic computing. Nat. Mater..

[34] Kawamoto D (2019). Correlation between Ni valence and resistance modulation on a SmNiO_3_ chemical transistor. ACS Appl. Electron. Mater..

[35] Massa NE (2015). Temperature and high-pressure dependent x-ray absorption of SmNiO_3_ at the Ni K and Sm L3 edges. Mater. Res. Express.

[36] Zhou Y (2016). Strongly correlated perovskite fuel cells. Nature.

[37] Shi J, Zhou Y, Ramanathan S (2014). Colossal resistance switching and band gap modulation in a perovskite nickelate by electron doping. Nat. Commun..

[38] Mansour AN, Melendres CA (1997). X-ray absorption spectra and the local structure of nickel in some oxycompounds and fluorides. J. Phys. IV Fr..

[39] Zhao L (2014). Multi-level control of conductive nano-filament evolution in HfO2 ReRAM by pulse-train operations. Nanoscale.

[40] Stathopoulos S (2017). Multibit memory operation of metal-oxide Bi-layer memristors. Sci. Rep..

[41] Du C, Ma W, Chang T, Sheridan P, Lu WD (2015). Biorealistic implementation of synaptic functions with oxide memristors through internal ionic dynamics. Adv. Funct. Mater..

[42] Goodman D, Brette R (2009). The brian simulator. Front. Neurosci..

[43] Lecun Y, Bottou L, Bengio Y, Haffner P (1998). Gradient-based learning applied to document recognition. Proc. IEEE.

[44] Diehl P, Cook M (2015). Unsupervised learning of digit recognition using spike-timing-dependent plasticity. Front. Comput. Neurosci..

[45] Kresse G, Furthmüller J (1996). Efficient iterative schemes for ab initio total-energy calculations using a plane-wave basis set. Phys. Rev. B.

[46] Blöchl PE (1994). Projector augmented-wave method. Phys. Rev. B.

[47] Kresse G, Joubert D (1999). From ultrasoft pseudopotentials to the projector augmented-wave method. Phys. Rev. B.

[48] Liechtenstein AI, Anisimov VI, Zaanen J (1995). Density-functional theory and strong interactions: Orbital ordering in Mott-Hubbard insulators. Phys. Rev. B.

[49] Persson, K. *Materials Data on SmNiO*_*3*_*(SG: 62) by Materials Project*. Dataset No. mp-25588 (2016).

[50] Zhang Z (2017). Perovskite nickelates as electric-field sensors in salt water. Nature.

[51] Zhang H-T (2019). Perovskite nickelates as bio-electronic interfaces. Nat. Commun..

[52] Kim S (2013). Physical electro-thermal model of resistive switching in bi-layered resistance-change memory. Sci. Rep..

[53] Mott, M. F. & Gurney, R. W. *Electronic Processes in Ionic Crystals* (Oxford Clarendon Press, 1948).

[54] Hooda MK, Yadav CS (2016). Electronic properties and the nature of metal–insulator transition in NdNiO3 prepared at ambient oxygen pressure. Phys. B Condens. Matter..

[55] Yan, H. et al. Multimodal hard x-ray imaging with resolution approaching 10 nm for studies in material sciences. *Nano Futures* **2**, 011001 (2018).

[56] Nazaretski E (2017). Design and performance of an X-ray scanning microscope at the Hard X-ray Nanoprobe beamline of NSLS-II. J. Synchrotron Radiat..

[57] Winarski RP (2012). A hard X-ray nanoprobe beamline for nanoscale microscopy. J. Synchrotron Radiat..

