# Supplementary material for: Perovskite neural trees
Source: Nat Commun. 2020 May 7;11:2245. doi: 10.1038/s41467-020-16105-y (PMC7206050; doi:10.1038/s41467-020-16105-y)
Supplement: Supplementary file 1 — Supplementary Information [file 41467_2020_16105_MOESM1_ESM.pdf]

Supplementary information

Perovskite neural trees

by Zhang et al.

This file includes:

- Supplementary Figures
- Supplementary Notes
- Supplementary References

Supplementary Figures

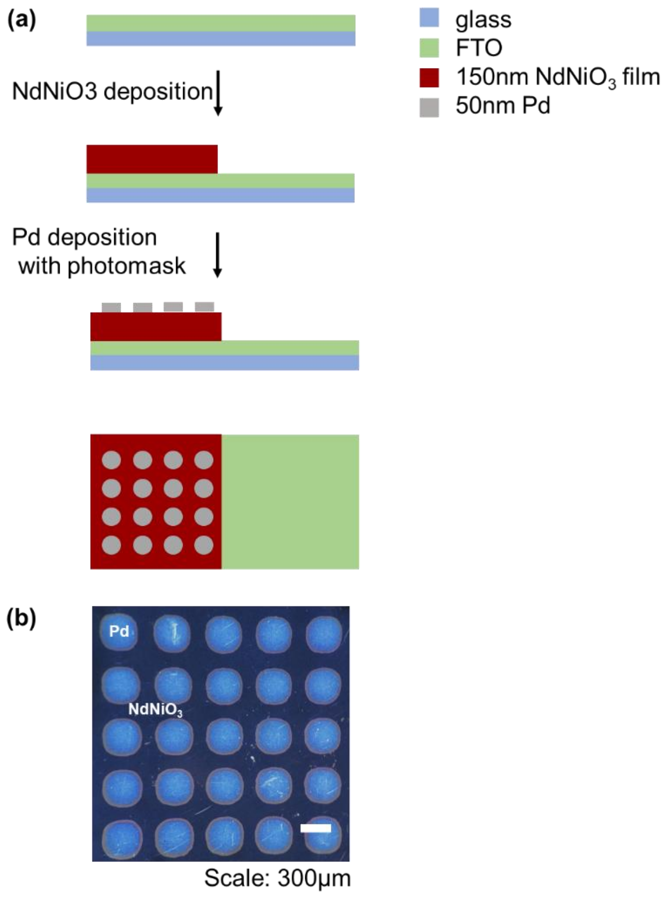

Supplementary Figure 1. Schematic figure of the fabrication process for vertical devices. (a) Detailed film growth and fabrication procedure can be found in Methods section**. (b)** An optical photograph of the device with Pd electrodes (lighter dots) on top of the $NdNiO_3$ film that is grown on FTO bottom electrode.

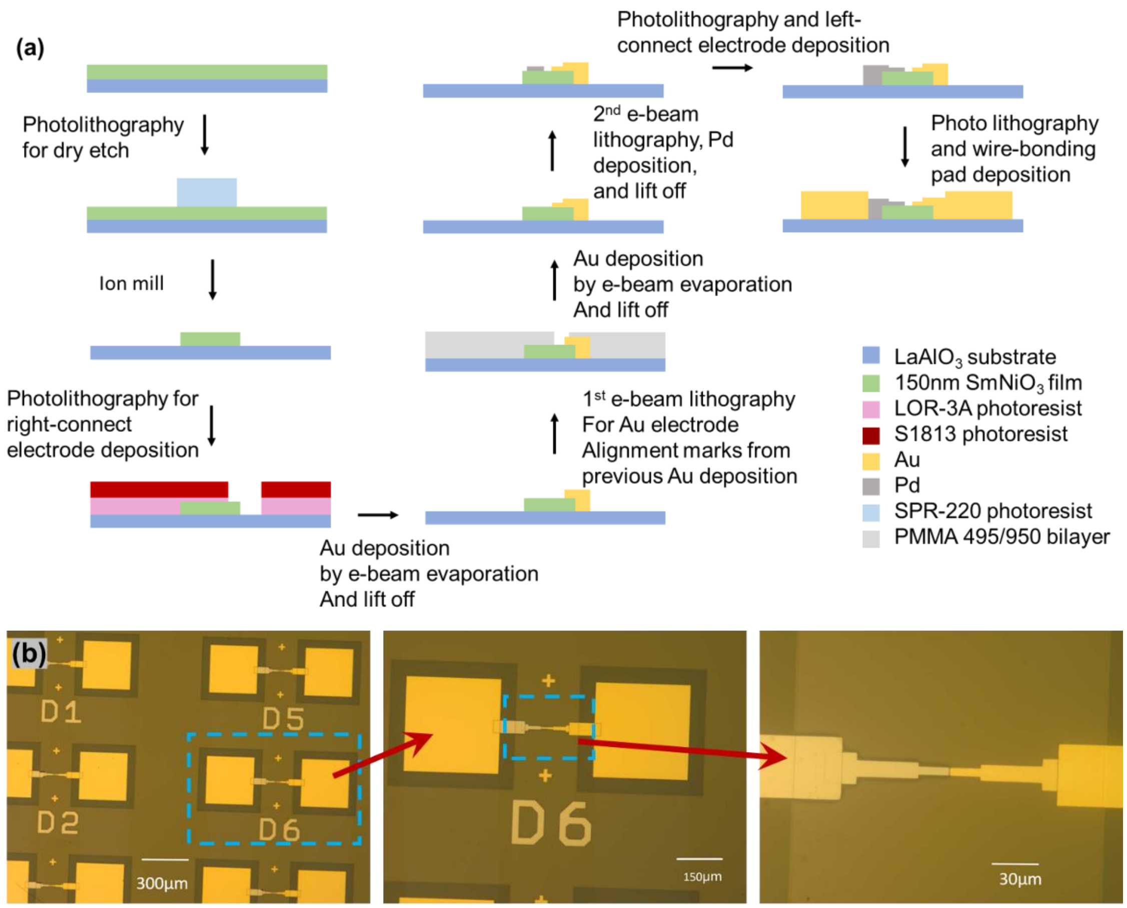

Supplementary Figure 2. Schematic figure of the fabrication process for in-plane devices. (a) Detailed film growth and fabrication procedure can be found in Methods section. **(b)** Optical images of the in-plane devices used for *in-situ* nano X-ray absorption spectroscopy measurements.

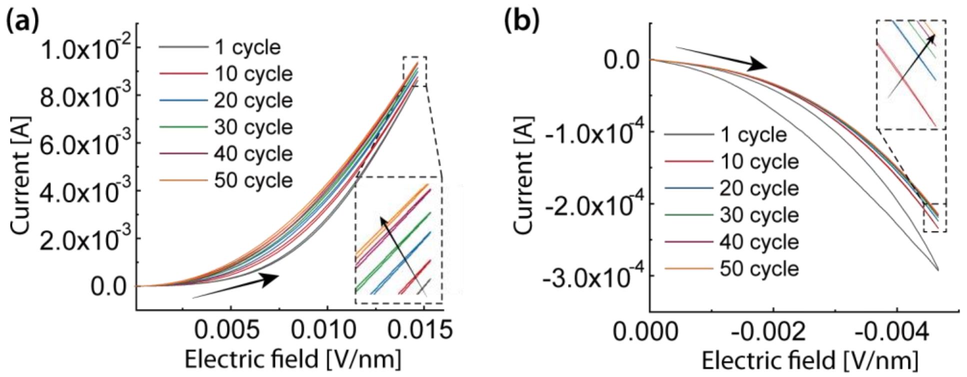

Supplementary Figure 3. I-V sweeping measurements of the nickelate device. (a) I-V characteristics under positive electric field (plotted every 10 cycles). The device resistance decreases continuously under positive field sweep. **(b)** I-V measurements from negative sweeps of the device and the device resistance increases continuously, indicating that overall device resistance can be modulated systematically by proton drift in the device. The arrow in each inset denotes sweeping direction.

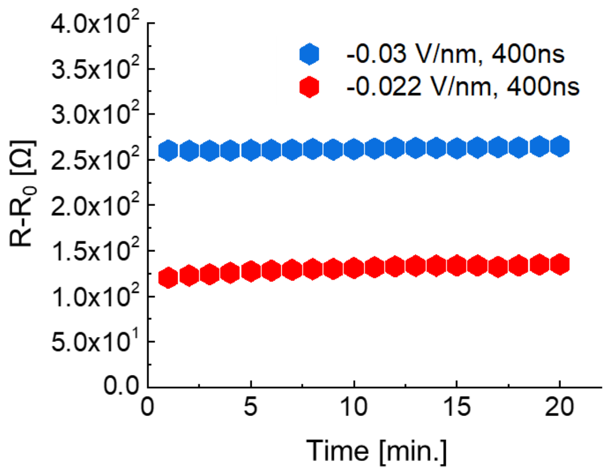

Supplementary Figure 4. Non-volatility of electrical states of the nickelate device. The device resistance was monitored as a function of time after application of a single electrical pulse, and the resistance change is non-volatile. Two representative data sets are shown.

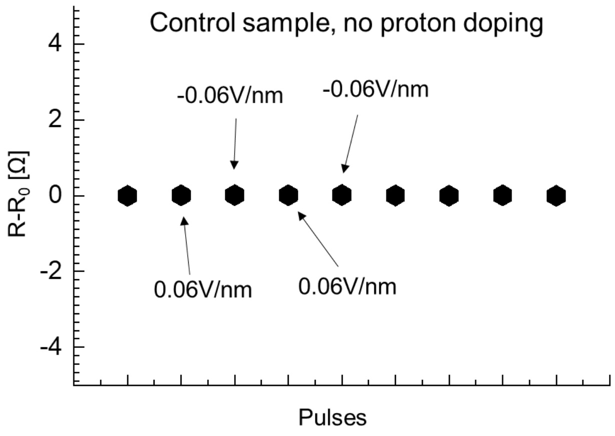

Supplementary Figure 5. Control experiment: Constant electrical resistance in pristine *un-doped* perovskite nickelate device under electric pulses. Periodic positive and negative pulses (0.06 V/nm, 1ms) were applied to a pristine perovskite nickelate device, and no resistance change was observed, indicating that the resistance change observed in the hydrogen doped devices is due to the proton motion under electrical pulses.

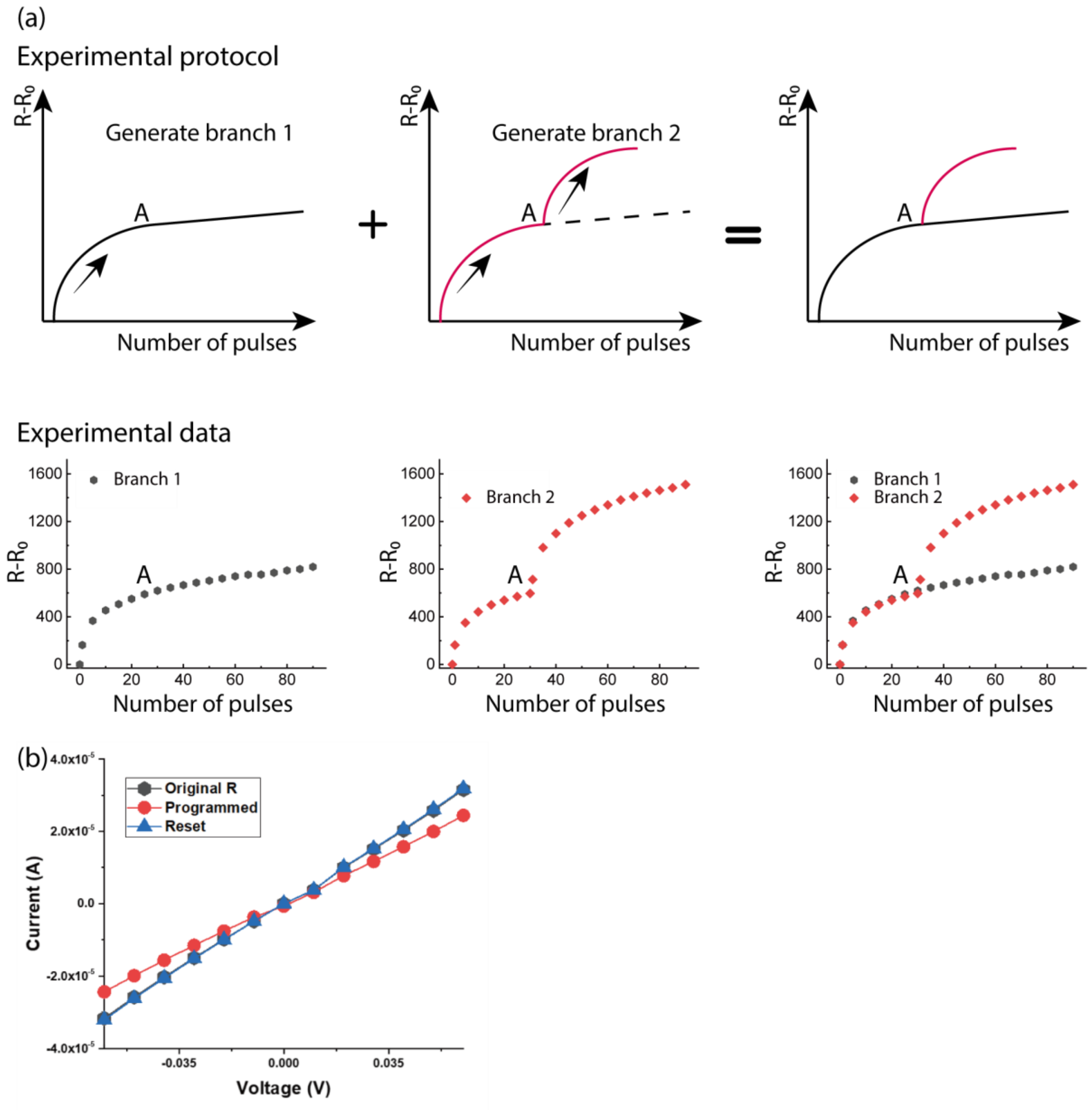

Supplementary Figure 6. Schematic of procedure for tree branch generation and measured experimental data. (a) Branch 1 is generated by applying consecutive constant electric field pulses to the device. To generated branch 2, the device is first reset to the original resistance state by applying an electrical pulse of the opposite polarity, as shown in (b); then consecutive pulses with the same pulse field as branch 1 is applied, and the device resistance change follows the same path to reach point A, at which point larger consecutive constant pulses are applied to generate a new branch 2. Similarly, multiple branches can be generated. Representative experimental data collected from our nickelate device is shown in the bottom figure. (b) A single reset pulse with opposite polarity (0.03 V/nm, 1 ms) is used to reset the device back to the original state from a programmed state.

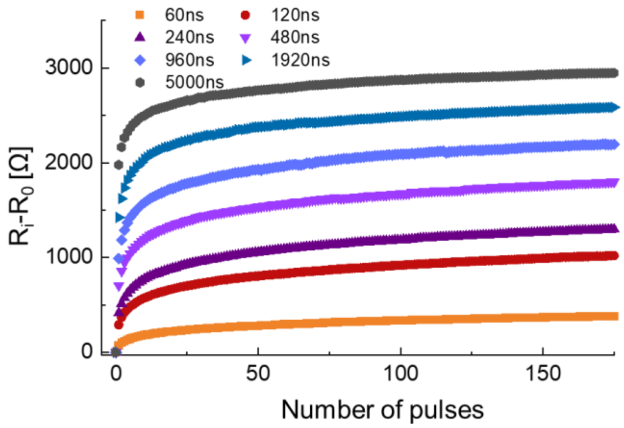

Supplementary Figure 7. Controlled synaptic weight updating. Controlled weight updating can be observed in the nickelate devices under consecutive pulses with multiple pulse widths (pulse field -0.027 V/nm). After ~175 pulses, the resistance change (R_{n+1}-R_n) is less than 0.15%. The saturation behavior of the synaptic strength for consecutive e-field pulses of same magnitude is similar to what is observed in biological synapses and is considered to be a crucial feature to maintain stability of neural circuits in the brain.[1,2]

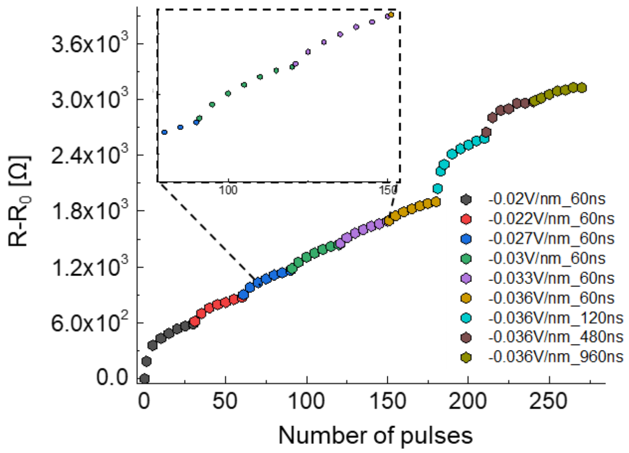

Supplementary Figure 8. Experimental data showing multiple generations of the tree branch structure. By increasing the stimulation pulse field and/or pulse width, the resistance branch can be generated over multiple generations, indicating sophisticated neural tree structures can be made possible with the perovskite nickelate devices. This is due to the synergistic combined effects of (a) sensitive dependence of the channel resistance to proton distribution due to charge localization as well as the (b) ability to control the migration of protons at near-atomic scale via electric fields.

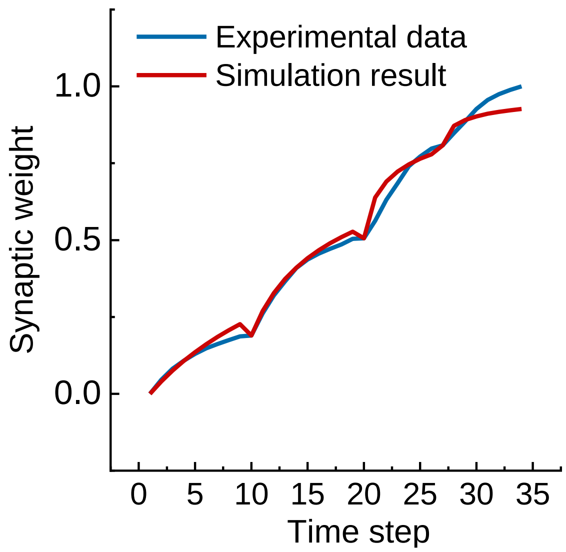

Supplementary Figure 9. Algorithmic simulation of nickelate device characteristics for object recognition. The experimentally obtained resistance curves are normalized between 0 to 1 for algorithmic interpretation of resistance as synaptic weight to be used in the neural network. This normalized curve is compared to weight change curves given by equation (1) for different u values. The input voltages which causes the resistance change in device are also appropriately scaled to the input for Δw curves for curve fitting. *Blue* curve represents the device resistance change (experiment) and simulated curve used for digit recognition is shown in *red*.

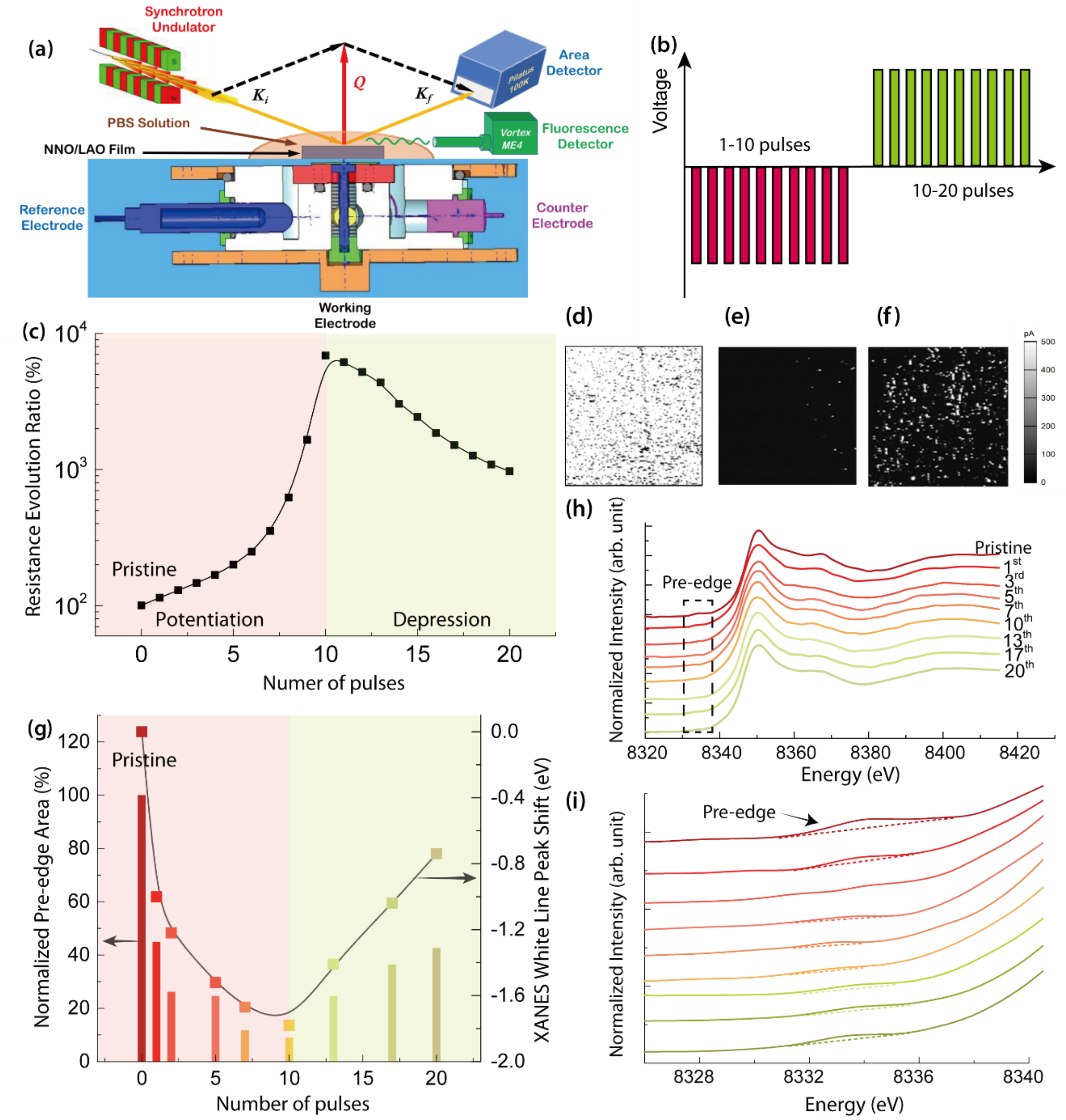

Supplementary Figure 10. Verification of the Ni valence change in XANES measurements using a macroscopic film region. (a) A schematic of the experimental setup for *in-situ* XAS measurement at 33ID-D in Advanced Photon Source (APS). A 10 mm × 10 mm $NdNiO_3$ thin film was connected to a working electrode and fixed onto a sample stage. 0.01 M PBS electrolyte was added on the film dropwise until fully covering the surface of the film. A Kapton film was then used to cover the electrolyte to avoid spillage during measurement. A Pt wire and customized Ag/AgCl electrode were also immersed in the electrolyte as counter and reference electrode. After each pulse treatment, the X-ray absorption spectroscopy signals were collected *in-situ*. **(b)** Schematic figure of how multiple pulses were applied on the nickelate film during potentiation (-500 mV, 30 s, 10 pulses) and depression (+500 mV, 30 s, 10 pulses). **(c)** Evolution of electrical resistance ratio (R/R_o×100%) during the potentiation and depression process. After the application of 10× potentiation pulses, the electrical resistance of film increased, suggesting the formation of insulating phase upon proton and electron uptake. When the bias with opposite polarity was applied, the resistance of $NdNiO_3$ film

decreased. **(d) (e)** and **(f)** A set of conducting atomic force microscopy (CAFM) images of $NdNiO_3$ before and after potentiation/depression pulses treatment. (d) for pristine sample, (e) for sample after potentiation and (f) for sample after depression. **(g)** The normalized pre-edge hump area evolution ($A/A_{pristine}$ ×100%) during *in-situ* treatment (arrow to the left), and the energy shift (vs. pristine $NdNiO_3$) of the white line peak of XANES spectra (arrow to the right). Upon potentiation, protons from the electrolyte were taken by the $NdNiO_3$ film and the Ni valence changed near-surface from Ni^{3+} to Ni^{2+}, leading to decrease of the pre-edge hump area as well as negative shift of the white line peak of XANES spectra. Upon bias application of reverse polarity, Ni^{2+} changed back to Ni^{3+} and the opposite trend was observed. **(h)** Raw data of the *in-situ* Ni K-edge XANES spectra. The box with dash line is the pre-edge region. **(i)** Zoom-in figure of pre-edge area of *in-situ* XANES spectra. This independent set of experiments conducted at the APS enables us to understand, verify and calibrate the XAS curves from pristine versus doped regions of the film, and aids in further understanding the nano-probe XAS experiments which requires great care in setting up and sample alignment.

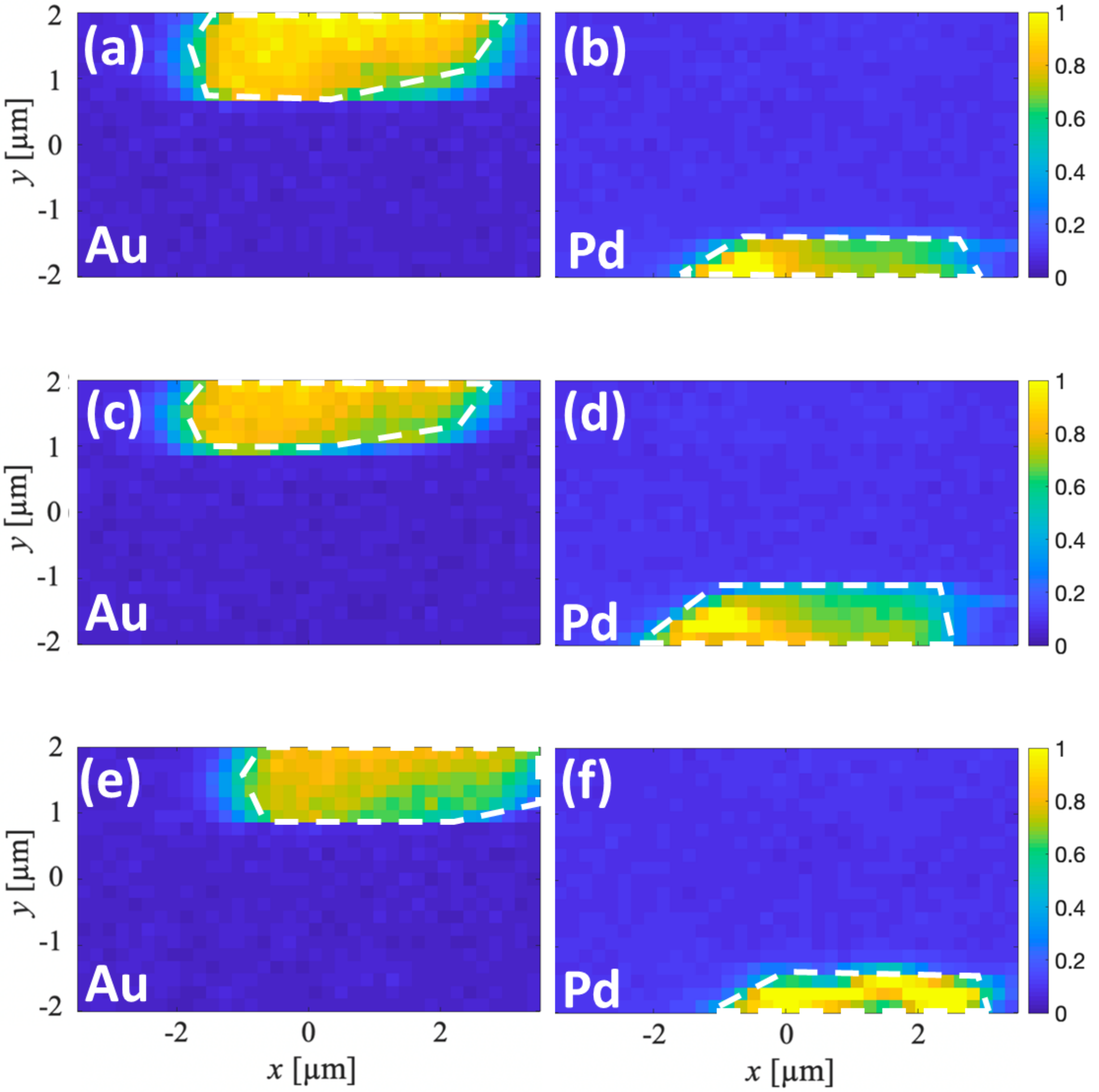

Supplementary Figure 11. Position determination of the Au and Pd electrodes. Fluorescence maps of the Au contact **(a, c, e)** and Pd contact **(b, d, f)** in the initial state **(a-b)**, after 2 mV pulses **(c-d)** and after 1V pulses **(c-d)**. Color indicates intensity of the fluorescence signal at M-edge of Au and L-edge of Pd. This set of experiments is used to calibrate the position of the electrodes during the XAS nano-mapping experiments.

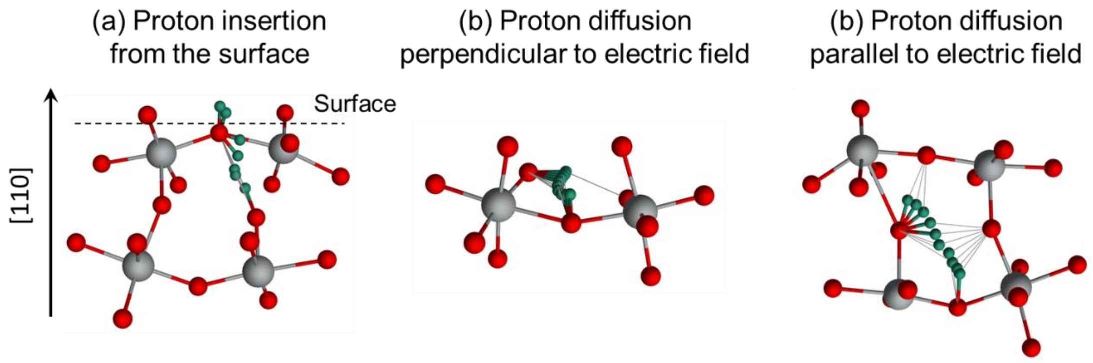

Supplementary Figure 12. Schematics of full pathways of proton diffusion. (a) proton insertion from surface **(b)** bulk diffusion pathway I **(c)** bulk diffusion pathway II. The proton, oxygen, and nickel atoms are described by the green, red and gray color balls. To visualize the complete pathway of proton migration we collected the protons from different images of NEB calculations and inserted into the initial images while keeping the positions of other atoms unchanged (i.e. same as in initial image) during proton hopping.

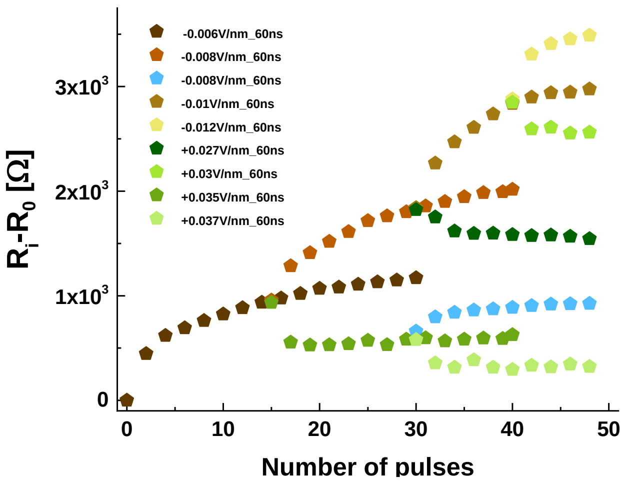

Supplementary Figure 13. Monolithic integration of perovskite synaptic devices on silicon platform. To demonstrate the potential compatibility of perovskite oxide-based devices with Si CMOS technology, an $NdNiO_3$ device was fabricated on Si substrate by thin film deposition and lithography. A 100 nm indium tin oxide (ITO) was first deposited on Si substrate as bottom electrode and the $NdNiO_3$ film thickness is 80 nm. Using the same method as described in Supplementary Figure 6, an ultrametric tree structure of memory states can be programmed by applying voltage pulses.

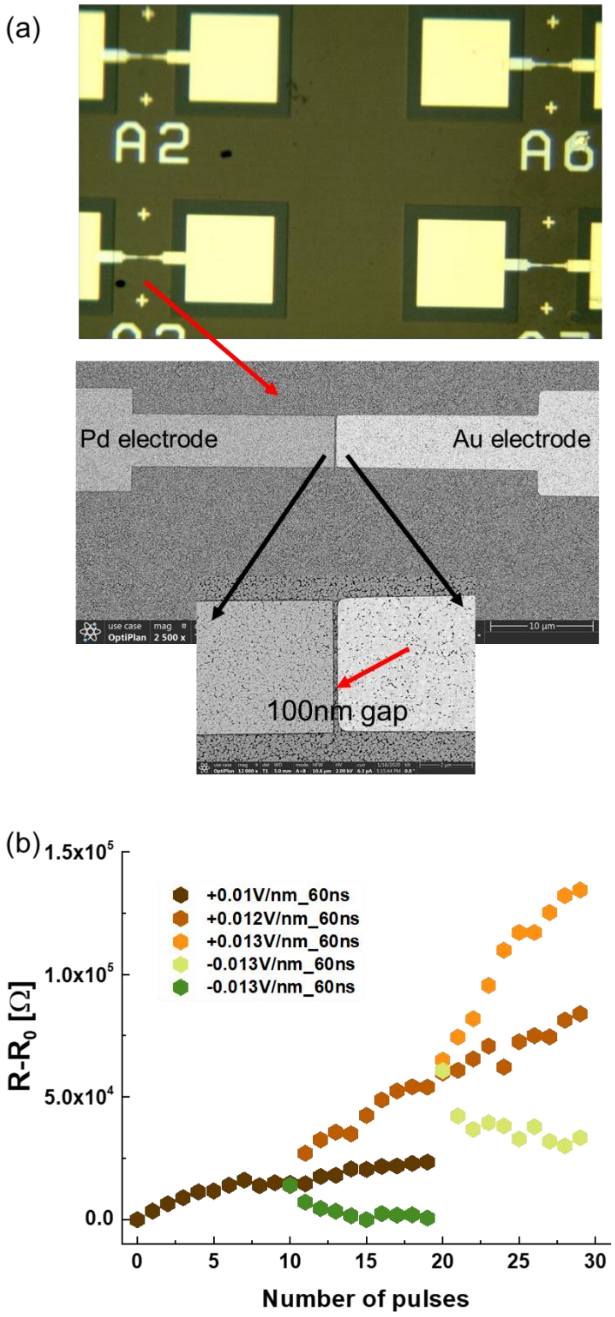

Supplementary Figure 14. Scalability of the perovskite device down to 100 nm scale. (a) Optical image and scanning electron microscope (SEM) image of $SmNiO_3$ devices with 100 nm gap size fabricated by lithography. **(b)** An ultrametric tree structure of memory states can be obtained by re-distribution of the protons through 60 nanosecond pulses. This provides proof-of-principle demonstration of synaptic behavior down to 100 nm channel device.

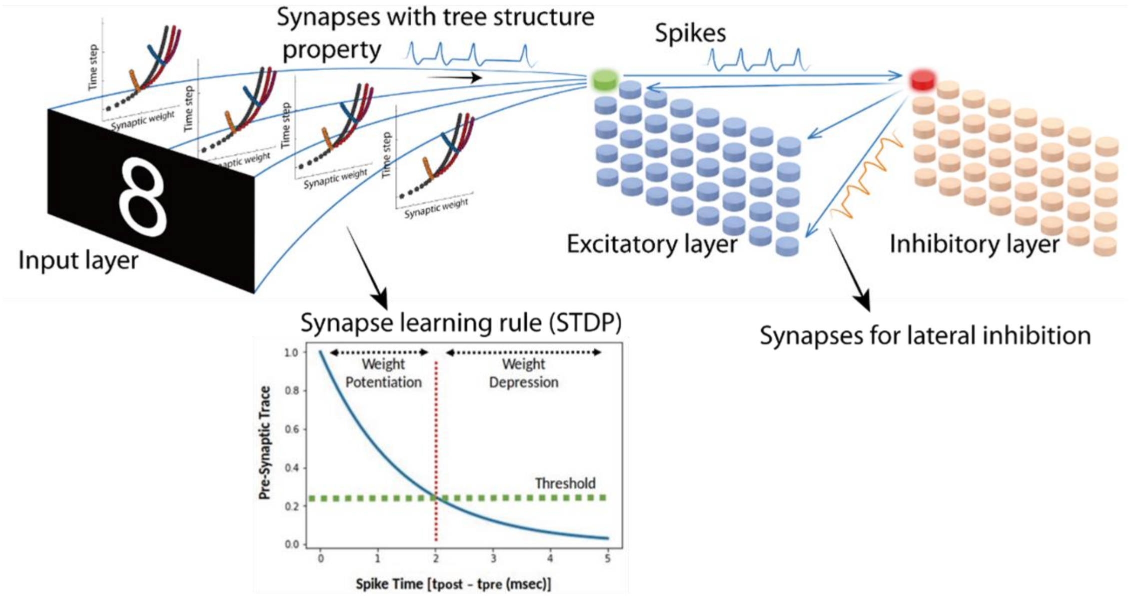

Supplementary Figure 15. The network architecture. Two-layer spiking neural architecture (with lateral inhibition and homeostasis) for object recognition.

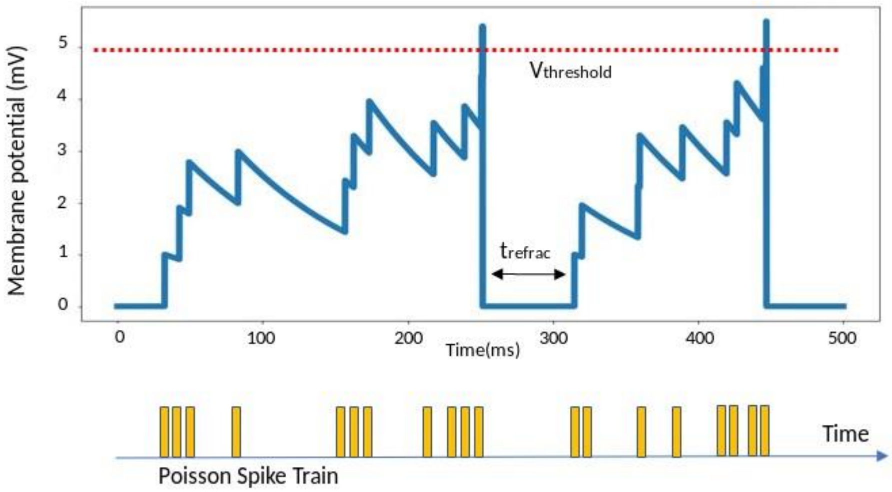

Supplementary Figure 16. Neuron dynamics of the neural network. Leaky integrate and fire neuron potential change shown by the plot of membrane potential against time.

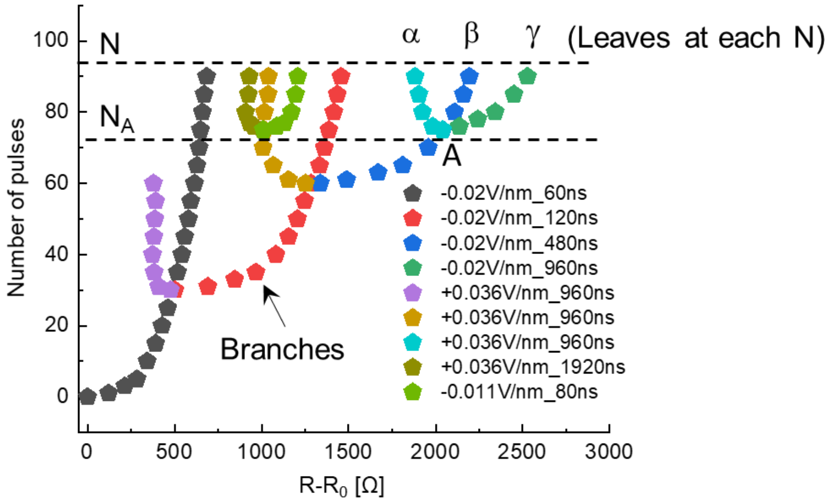

Supplementary Figure 17. A perovskite ultrametric neural tree with *K*=3. The tree is experimentally obtained by applying electrical pulses at room temperature. The experimental data shown in this figure were collected from the identical device discussed in **Figure 2** of main manuscript.

Supplementary Notes

Supplementary Note 1. Network Architecture

The network architecture used in this work is a two-layer spiking neural network, as shown in **Supplementary Figure 15**. Each neuron is assigned to each pixel of the input image. Depending on the pixel intensity value, the neuron outputs a Poisson distributed spike train. The duration of Poisson distributed spike train in our simulation was 350ms. One millisecond corresponds to one single time-step for the simulations. Therefore, 350ms duration is equal to 350 time-steps. Excitatory layer receives spikes from input layer and depending on the neuron model the membrane potential of these neurons changes. Excitatory layer neurons are connected to input layer via synapses. These synapses propagate the spikes from input layer to excitatory layer and also changes its strength depending on the synapse learning rule. Each inhibitory neuron receives connection from one neuron from excitatory layer and connects back to all other excitatory neurons. The number of inhibitory neurons is equal to number of excitatory neurons. The purpose of this layer is to provide lateral inhibition for competitive learning.

1.2 Neuron Model

Leaky-integrate-and-fire (LIF) neuron model is used as the excitatory neuron. The differential equation form of this neuron model is as following,

$$\tau \frac{\partial V_{mem}}{\partial t} = (E_{rest} - V) + g_e(E_{exe} - V) + g_i(E_{inh} - V)$$

τ is membrane potential decay constant, V_{mem} is membrane potential of neuron, E_{rest} is resting membrane potential, conductance values for excitatory and inhibitory synapses are g_e and g_i, E_{exe} and E_{inh} are equilibrium potential of excitatory and inhibitory synapses.

$$\tau_{g_i} \frac{\partial g_i}{\partial t} = -g_i$$

A dynamic conductance change model was used for synapses. i.e., when a pre-synaptic neuron fires, the synaptic conductance instantaneously changes according to their strengths and then decays exponentially with a time constant.[3] So, if pre-synaptic neuron is inhibitory in nature and spikes, then the conductance g_i of synapse is updated. To have direct control over membrane potential, g_e of excitatory neuron is kept zero. Whenever a pre-synaptic spike occurs, if pre-synaptic neuron is excitatory then membrane potential of post-synaptic neuron is updated directly and if pre-synaptic neuron is inhibitory, then g_i is updated. τ_{gi} is the time constant of inhibitory post-synaptic potential. As the membrane potential of neuron increases with the incoming spikes, it generates spikes if the membrane potential reaches its threshold value V_{thresh} and becomes in-active for certain time period t_{refrac} i.e, membrane potential is reset to the resting potential V_{rest} and neuron's membrane potential does not change during this time t_{refrac}. These neuron dynamics are illustrated in **Supplementary Figure 16**.

1.3 Synapse learning

Spike time dependent plasticity (STDP) is used as learning rule for synapses. The synapses maintain two parameters, first is its weight (strength) and second is the spike trace. Spike trace keeps track

of spiking activity of a pre-synaptic neuron. Value of trace is updated by 1 whenever there is pre-synaptic spike and it decays exponentially.

$$\Delta\omega = \eta(x_{pre} - x_{tar})(\omega_{max} - \omega)^{\mu}$$

x_{pre} is the pre-synaptic trace and x_{tar} is the threshold of trace. When x_{pre} is greater than x_{tar} , it will cause potentiation and x_{pre} less than x_{tar} will cause depression. w_{max} is maximum weight which can be attained by synapse and 'η' is learning rate.

$(w_{max} - w)^u$ factor ensures that the amount of change in synapse weight saturates and it approaches w_{max}, thereby acting as weight controlling factor. The exponent u controls the rate of saturation as the weight change occurs. Higher u corresponds to slow weight saturation i.e, higher u forces small changes to synapse weight, therefore it takes longer to reach w_{max}.

1.4. Training and Testing of Spiking neural network

1.4.1 Training:

Spiking neural network (SNN) is trained on the Modified National Institute of Standards and Technology database (MNIST) dataset.[4] The MNIST dataset has a collection of total 70000 grey scale images of single digits. Each image is 28×28 pixel data. 60000 images are used for training the network and 10000 for testing the network's prediction accuracy. Spike time dependent plasticity rule (STDP) rule is used as unsupervised learning rule. Each training image is shown to the network for 350 timesteps. Each timestep represents 1ms, therefore total 350ms. The input image pixel values are converted to Poisson spike train in the input layer. These spike trains are then propagated to excitatory layer neurons through synapses. The potential of excitatory neurons (V_{mem}) increases as they receive spikes and once the potential reaches threshold ($V_{threshold}$) the neuron spikes. The weight of all the synapses connected to the neuron which spiked are updated. This update happens using STDP learning rule which in turn uses the pre-synaptic neuron's spiking activity trace x_{pre}. If the pre-synaptic neuron trace is greater than x_{tar}, then the synapse is potentiated, else synaptic depression occurs. Synaptic weights are always updated when a post-neuron spikes *i.e,* whenever an excitatory neuron spikes. The inhibitory neuron receives spikes from an excitatory neuron and connects back to all other neurons in excitatory layer. This is done to encourage competitive learning between neurons. So whenever an excitatory neuron's spiking activity increases, it causes inhibitory neuron to spike and these inhibitory spikes reduces the membrane potential of other neurons, thus causing a decrease in spiking activity of other neurons.

1.4.2 Testing:

At the end of training, each neuron is assigned a tag of a digit *i.e,* each excitatory neuron now represents a digit. So if the number of spikes generated by a particular neuron is more than all the other neurons when a testing digit is shown to the network, then the tag represents the digit that is recognized. The processes of identifying the digits to be assigned to each excitatory neuron is started when we are 5000 images away from completing training of the network. During this period, number of spikes of each neuron for each image is stored. After every 500 images, all the neurons are assigned digits and this occurs for the final 5000 images. For every 500 images shown, the spiking rate of each neuron for each digit is sampled and the neuron with maximum spiking rate or group of neurons whose spiking rate is above a certain threshold are assigned the digit tag. For

example, to assign digit 9 to excitatory neurons, for the 500 images shown, number of images with digit 9 are sampled and the spiking activity of neurons belonging to those images is averaged to obtain spiking rate of each neuron. Now, among these neurons, those of which are above a threshold spiking rate for digit 9 are assigned a tag of digit 9. Recurring assignments are done over last 5000 images so that a generalized digit tag is assigned to each excitatory neuron. 10000 test images provided by MNIST dataset was used to determine the accuracy of the network. When we test the network, the learning is frozen *i.e,* no synaptic weight updates are performed when we pass an image to the network. The testing image is input to the network and excitatory neuron which spikes highest number of times is identified and the digit tag belonging to that neuron indicates the recognized digit.

Supplementary Note 2. Perovskite Ultrametric Trees and Spin Glasses

Here, we explain the apparent connection between the experimental voltage-resistance curves reported in the manuscript and magnetization-temperature curves reported for spin glasses. The notion of a spin glass originated with the study of the low temperature state of substitutional magnetic alloys, with finite concentrations of magnetic ions in non-magnetic hosts.[5–8] In general, spin glasses are models characterized by disorder and frustration. Disorder implies that interactions between different states of the system are random. Frustration usually means that conflicting interactions compete with each other and consequently the system doesn't settle on a single equilibrium state satisfying all constraints, but rather a multitude of equilibrium states. In experiments, tree states in spin glasses have been typically accessed by changing the global temperature (heating-cooling cycles) and the corresponding experimental data have been reported at very low temperatures in the 1 – 20 Kelvin range, such as for CuMn and $CoCl_2$ systems.[9–12] In the context of the present study, by inserting impurity dopants in the form of hydrogen and subjecting the resulting material to a bias voltage, one obtains a regime exhibiting certain characteristics that is prototypical of a spin glass. Here, the *voltage* will play the role of the "temperature" and the *resistance* of the material will define a "state" of the system. The data measured from our nickelates (see the figure below) forms a tree whose branching ratio is given by $K = 3$:

This defines an *ultrametric* topology on the space of states – a characteristic feature of spin glasses – in the following way. Fix N, the number of pulses, and let Σ_N be the collection of all states corresponding to N. These correspond to the extremities (the leaves) of the branches at level N. For any two states $\alpha,\beta \in \Sigma_N$, let A denote the closest common ancestor (see **Supplementary Figure 17** for an illustration) and let N_A be the corresponding number of pulses. The *overlap* $R_N(\alpha,\beta)$ is given by

$$R_N(\alpha,\beta) = N_A/N.$$

The lower one must go to find this ancestor, the smaller the overlap. The distance $d_N(\alpha,\beta)$ between α and β is then defined as

$$d_N(\alpha,\beta) = 1 - R_N(\alpha,\beta),$$

which can be viewed as the normalized depth of the common ancestor A. It has the property that for any three states, α,β,γ in Σ_N, at least two of the distances $d_N(\alpha,\beta)$, $d_N(\beta,\gamma)$, $d_N(\alpha,\gamma)$ are equal. In the case when exactly two distances are equal, the third is shorter. For instance, in Figure 4, these distances are equal to each other since they all share the same common ancestor A. The space (Σ_N,d_N)

satisfying this property is said to be ultrametric.[5] This allows a hierarchical structure on the state space by grouping all states within a certain distance into a single cluster. Then it is straightforward to see that ultrametricity implies that these clusters partition the space with no overlapping among different clusters. The theoretical development of spin glass phase and their potential use in neural networks has a long history and is an active area of research.[6,13] The experiments reported in this paper presents a physical realization of such models at room temperature allowing a hierarchical structure of arbitrary level *N* and branching ratio *K* that can be accessed electrically in solid state devices at ambient conditions and in a reversible manner.

Supplementary References

1. Turrigiano, G. G. & Nelson, S. B. Homeostatic plasticity in the developing nervous system. *Nat. Rev. Neurosci.* **5**, 97–107 (2004).

2. Zhuang, X. *et al.* Hyperactivity and impaired response habituation in hyperdopaminergic mice. *Proc. Natl. Acad. Sci. U. S. A.* **98**, 1982–1987 (2001).

3. Diehl, P. & Cook, M. Unsupervised learning of digit recognition using spike-timing-dependent plasticity. *Front. Comput. Neurosci.* **9**, 99 (2015).

4. Lecun, Y., Bottou, L., Bengio, Y. & Haffner, P. Gradient-based learning applied to document recognition. *Proc. IEEE* **86**, 2278–2324 (1998).

5. Rammal, R., Toulouse, G. & Virasoro, M. A. Ultrametricity for physicists. *Rev. Mod. Phys.* **58**, 765–788 (1986).

6. Sherrington, D. Spin glasses: a perspective. in *Spin Glasses* 45–62 (Springer, 2007).

7. Edwards, S. F. & Anderson, P. W. Theory of spin glasses. *J. Phys. F Met. Phys.* **5**, 965–974 (1975).

8. Mezard, M., Parisi, G. & Virasoro, M. *Spin Glass Theory and Beyond*. *World Scientific Lecture Notes in Physics* vol. Volume 9 (WORLD SCIENTIFIC, 1986).

9. Nagata, S., Keesom, P. H. & Harrison, H. R. Low-dc-field susceptibility of CuMn spin glass. *Phys. Rev. B* **19**, 1633–1638 (1979).

10. Matsuura, M., Tanaka, N., Karaki, Y. & Murakami, Y. Related content Spin Glass-like Anomalous Memory in Ferromagnetic MCl2-GIC3(M:Co,Ni). *Japan Soc. Appl. Phys.* **26**, 797–798 (1987).

11. Suzuki, I. S. & Suzuki, M. Effect of random disorder and spin frustration on the reentrant spin-glass and ferromagnetic phases in the stage-2 Cu0.93Co0.07Cl2 graphite intercalation compound near the multicritical point. *Phys. Rev. B* **73**, 1–11 (2006).

12. Suzuki, M., Suzuki, I. S. & Matsuura, M. Memory and aging effect in hierarchical spin orderings of the stage-2 CoCl2 graphite intercalation compound. *Phys. Rev. B* **73**, 1–13 (2006).

13. Talagrand, M. Mean field models for spin glasses. in *A Series of Modern Surveys in Mathematics* (Springer-Verlag, 2011).
